# Beware of False “Type Strain” Genome Sequences

**DOI:** 10.1128/MRA.00369-19

**Published:** 2019-05-30

**Authors:** Francisco Salvà-Serra, Daniel Jaén-Luchoro, Roger Karlsson, Antoni Bennasar-Figueras, Hedvig E. Jakobsson, Edward R. B. Moore

**Affiliations:** a Department of Infectious Diseases, Institute of Biomedicine, Sahlgrenska Academy, University of Gothenburg, Gothenburg, Sweden; b Clinical Microbiology, Sahlgrenska University Hospital, Gothenburg, Sweden; c Culture Collection University of Gothenburg, Sahlgrenska Academy, University of Gothenburg, Gothenburg, Sweden; d Centre for Antibiotic Resistance Research (CARe), University of Gothenburg, Gothenburg, Sweden; e Microbiology, Department of Biology, University of the Balearic Islands, Palma de Mallorca, Spain; f Nanoxis Consulting AB, Gothenburg, Sweden; g Area of Infectious Diseases, Research Institute of Health Sciences (IUNICS-UIB), University of the Balearic Islands, Palma de Mallorca, Spain; Indiana University, Bloomington

## LETTER

With this letter, we warn users of bacterial DNA sequence data about recent cases misusing the term “type strain” in bacterial genome sequence reports and highlight the importance that the term is used in the correct context.

In recent articles published in the journal *Genome Announcements* (GA), (now *Microbiology Resource Announcements* [MRA]) (1–5), the complete genome sequences of five strains of five species of bacteria were reported. The titles of the articles stated that the strains represent the respective “type strains” of the five species, although the articles do not present details about the processes of defining the type strains. At this point, it is important to point out that the concept and description of “type strain” are not arbitrary; the type strains of bacterial species are defined by rule 18a of the International Code of Nomenclature of Prokaryotes as follows: “The type strain is made up of living cultures of an organism, which are descended from a strain designated as the nomenclatural type” (6). Strains serving as nomenclatural type material are designated precisely, and this may be done in various ways (7); typically, a “holotype strain” is designated at the time of valid publication of a new species name (rule 18b), in which the type strain must conform to defined conditions (rule 30). The five species described in the GA publications had been validly published previously, and the type strains for those species were already defined, preserved, and publicly available in numerous culture collections. Meanwhile, the strains reported in the five GA articles were recently isolated independently. Therefore, since they are not descended from the already defined type strains, they cannot represent the authentic type strains of the species.

To confirm these observations, we performed average nucleotide identity (ANI) analyses using JSpeciesWS (8, 9) between the five genome sequences reported in the GA articles and the genome sequences of the documented type strains of the five species (Table 1). The ANI values range from 83.10 to 98.96% (percentages aligned, 75.56 to 91.18%), confirming that the described strains are different and, furthermore, confirming that one of them, Lelliottia nimipressuralis SGAir0187, is misclassified and is not a strain of this species.

**TABLE 1:** Average nucleotide identity values between the genome sequences of the strains erroneously reported as type strains and the genome sequences of the authentic type strains of the corresponding species

Strain erroneously reported as type strain	GenBank accession no.	Authentic type strain	GenBank accession no.	ANIb^*a*^ (%)	Aligned nucleotides (%)
Acinetobacter indicus SGAir0564	CP024620	*A. indicus* CIP 110367^T^	AYET00000000	96.02	83.59
Bacillus altitudinis SGAir0031	CP022319	*B. altitudinis* 41KF2b^T^	ASJC00000000	97.92	91.18
Lelliottia nimipressuralis SGAir0187	CP025034	*L. nimipressuralis* CCUG 25894^T^	SDDX00000000	83.10	75.56
Pseudomonas stutzeri SGAir0442	CP025149	*P. stutzeri* CGMCC 1.1803^T^	CP002881	97.04	88.04
Staphylococcus haemolyticus SGAir0252	CP025031	*S. haemolyticus* NCTC 11042^T^	PPQA00000000	98.96	88.85

a Average nucleotide identity based on BLAST. Species threshold, ≥95%.

Representing genome sequences from false type strains of given species has the potential to lead to erroneous conclusions in future studies that may rely on the published misinformation, as they may be used as the wrong reference points. We encourage the authors of the five GA publications and the journal to publish *corrigenda* and remove the words “type strain” from the titles of the publications. We also warn users of publicly available genome sequence data to be cautious in accepting the metadata associated with genome sequences; recent studies have clearly demonstrated the presence of high numbers of misclassified genome sequences in different taxa according to genome sequence identity criteria for species-level identifications (10–13). Public databases archiving genome sequence data are implementing new controls for catching sequence deposits that do not adhere to accepted taxonomic standards for identification. However, the public databases may be able to control descriptions of only the sequence data submitted; they cannot control the descriptions of the data that authors may use in publications. We encourage users of reported and archived genome sequence data to perform relevant taxonomic controls, for instance, by comparing housekeeping gene sequences from genomes with independently determined sequences of documented type strains; if possible, comparisons should be made from the data of the original descriptions of the species.

In conclusion, type strains serve as important “reference points” in bacterial taxonomy and systematics and are fundamental and essential resources for microbiology. The presence of sequences erroneously reported as type strains is a real example of “fake news” and is potentially dangerous, as it can easily lead to further errors in studies relying on them. With the presented examples of inaccurate and inappropriate use of the term “type strain,” we emphasize how important it is to confirm the designation. In fact, we would propose that the public databases establish dedicated repositories for the genome sequences of the type strains of validly published species. Essentially, while genome sequence data represent enormous resources, it is imperative to perform basic and relevant controls when reporting and using public genome sequences.
